# The Relative Contributions of *BmPPO* and *BmDDC* in Immune Melanization of Hemolymph in Silkworm, *Bombyx mori*

**DOI:** 10.3390/insects17040405

**Published:** 2026-04-09

**Authors:** Zunmei Hu, Pan Chen, Chunyang Wang, Ping Chen

**Affiliations:** 1 State Key Laboratory of Resource Insects, Southwest University, Chongqing 400715, China; 15054952371@163.com (Z.H.);; 2 College of Sericulture, Textile and Biomass Sciences, Southwest University, Chongqing 400715, China

**Keywords:** phenoloxidase, dopa decarboxylase, immune melanization, *Bombyx mori*

## Abstract

Lepidoptera constitute a major group of agricultural and forestry pests. Therefore, investigating the immune mechanisms of the model species *Bombyx mori* may provide valuable insights for the development of improved pest management strategies. In insects, phenoloxidase (PO) and dopa decarboxylase (DDC) in immune melanization have been widely studied individually, yet their relative contributions have rarely been investigated. Here, we demonstrate that pharmacological inhibition of either PO or DDC in *Escherichia coli*-infected larvae significantly suppresses hemolymph melanization, with PO inhibition causing a more pronounced reduction than DDC inhibition. Consistently, RNA interference-mediated knockdown of *BmPPO1* or *BmPPO2* markedly decreased hemolymph melanization following infection. This results in both PO and DDC contributing to immune-induced hemolymph melanization, with PO playing a dominant role in this process. In contrast, compared to PO inhibition, DDC inhibition leads to significant damage to hemolymph antibacterial activity and cellular immune responses, including hemocyte aggregation, encapsulation, and phagocytosis. In addition, compared with the knockdown of *BmPPO1* or *BmPPO2*, the knockdown of *BmDDC* leads to a more severe decrease in antibacterial activity and cellular immune function. Exogenous addition of dopamine can partially rescue cell damage, indicating that both DDC and PO play a role in cellular immunity, but DDC has a slightly stronger effect. Overall, this study provides important insights into the immunity of hemolymph in insects and other arthropods.

## Simple Summary

Melanization is an important immune defense mechanism in arthropods, and hemolymph melanization represents a conserved immune response. Phenoloxidase (PO) and dopa decarboxylase (DDC) are known to participate in immune melanization, but their relative contributions remain unclear. In this study, we inhibited PO activity using phenylthiourea and suppressed DDC activity using carbidopa to examine their effects on hemolymph melanization, antibacterial activity, hemocyte aggregation, encapsulation, and phagocytosis during the immune response. In addition, RNA interference was used to reduce the expression of *BmDDC*, *BmPPO1*, and *BmPPO2* to further investigate their roles in hemolymph melanization, antibacterial activity, hemocyte aggregation, and nodulation. Our results show the relative contributions of PO and DDC in melanization and immune defense in infected hemolymph, respectively.

## 1. Introduction

Melanization is a hallmark innate immune response uniquely conserved in arthropods. Upon infection or tissue injury, melanization is rapidly activated to produce melanin at the site of insult, thereby confining invading microorganisms and facilitating wound repair [1,2,3,4,5,6]. In addition to its immune function, melanization plays a critical role in cuticular sclerotization, a developmental process that hardens the insect exoskeleton and constitutes the first physical barrier against pathogen invasion [4,5,6,7,8,9]. Melanin synthesis is driven by a series of enzyme-catalyzed reactions that convert phenolic and other aromatic compounds into 5,6-dihydroxyindole (DHI), which subsequently polymerizes to form melanin [2,5,10,11,12,13,14,15]. Phenoloxidase (PO) exhibits broad substrate specificity, including monophenols and o-diphenols such as tyrosine, 3,4-Dihydroxyphenyl (L-DOPA), and dopamine, and plays a crucial role in melanin formation and immune defense [3]. Extensive studies have demonstrated that PO participates in multiple immune-related processes, including cuticular sclerotization, phagocytosis, encapsulation, and nodule formation [3,6,8,16,17]. In insects, PO typically exists as an inactive zymogen known as prophenoloxidase (proPO or PPO), which requires proteolytic cleavage for activation [16,17,18]. Upon immune challenge, a serine protease cascade is triggered, leading to the conversion of PPO into active PO and initiating the melanization response [18,19,20]. The number of PPO genes varies substantially across species, ranging from one in *Apis mellifera* to ten in *Aedes aegypti*, reflecting lineage-specific expansion and potential functional diversification [21,22,23,24,25,26].

In parallel, dopa decarboxylase (DDC) catalyzes the decarboxylation of DOPA to form dopamine, which is a key step in melanin synthesis. DDC expression peaks immediately before or after molting, and RNA interference (RNAi) of DDC during the fourth larval instar results in albino pupae and adults in *Henosepilachna vigintioctopunctata* [17]. DDC enzymatic activity is required for cuticular tanning and melanization in *Manduca sexta* and *B. mori* [15,27]. Silencing DDC significantly reduces melanization of *Dirofilaria immitis* microfilariae in the hemocoel of *Armigeres subalbatus*, resulting in increased parasite survival [28]. In crustaceans and insects, DDC levels are closely correlated with hemocyte abundance, phagocytic activity, pathogen clearance efficiency, and host survival following bacterial infection [29,30,31]. Moreover, functional studies in the medfly *Ceratitis capitata* using siRNA, antibodies, and pharmacological inhibitors have demonstrated that hemocyte aggregation, melanization, phagocytosis, and nodulation are dependent on DDC activity [32]. Together, these studies suggest that DDC and PO participate in overlapping immune processes, particularly in melanization-associated defenses. However, studies investigating their characteristics in functional roles remain limited.

In insects and other arthropods, the open circulatory system places hemolymph at the center of immune surveillance and defense, and multiple pathogens are used to stimulate immune melanization, among which *Escherichia coli* is used as a safe and efficient pathogen [33,34,35,36,37,38]. RNAi is a conserved gene silencing mechanism in which double-stranded RNA (dsRNA) is processed into siRNAs that guide sequence-specific degradation of target mRNA [39,40]. Widely used for functional genomics, RNAi enables efficient investigation of gene function in diverse organisms, including *Bombyx mori* [41]. Using the silkworm *B. mori*, an economically important lepidopteran model, we examined melanization responses, bacterial proliferation, and cellular immune reactions in infected hemolymph. Our study revealed that DDC and PO play overlapping roles in hemolymph melanization and immune defense, although their relative contributions differ. These findings provide new insights into the mechanisms underlying immune melanization in insects and other arthropods.

## 2. Materials and Methods

### 2.1. Experimental Silkworm

The silkworm (*B. mori*) strain D9L was maintained at Southwest University (Chongqing, China) under standard rearing conditions (24–26 °C, 70–85% relative humidity, and a 12 h light/12 h dark photoperiod) and fed fresh mulberry leaves. Larvae of day-1 fifth instar were chosen based on similar size in the experiments.

### 2.2. Pathogen and Inhibitor Preparation

The *E. coli* strain BL21 constitutively expressing enhanced green fluorescent protein (EGFP) was cultured overnight at 37 °C in Luria–Bertani (LB) medium with shaking. Cells were harvested by centrifugation at 8000× *g* for 10 min at 4 °C, washed twice with phosphate-buffered saline (PBS; 8 g NaCl, 0.2 g KCl, 1.44 g Na_2_HPO_4_, 0.24 g KH_2_PO_4_ per liter, pH 6.5), and resuspended in PBS to an optical density of OD_600_ = 0.8.

Phenylthiourea (PTU, catalog No. P7629, Sigma-Aldrich, St. Louis, MO, USA) and Carbidopa (catalog No. C1330, Sigma-Aldrich, St. Louis, MO, USA) were separately dissolved in absolute ethanol to prepare stock solutions at a concentration of 27 mM. The stock solutions were sealed and stored at 4 °C, protected from light and diluted with PBS (pH 6.5) to a final concentration of 2 mM immediately before use.

### 2.3. Silkworm Injection and Hemolymph Collection

Select larvae with healthy phenotypes for injection or collection of hemolymph.

Larvae were cold anesthetized and were then injected with liquid using a homemade capillary syringe through the spiracle into the hemocoel to avoid bleeding as much as possible. Inject inhibitor solution (inhibitor) or dsRNA solution (dsRNA) through the right stomata of larvae, and inject *E. coli* suspension (*E. coli*) through the left stomata of larvae. The injection volume is 5 μL.

Larve was surface-sterilized with 75% alcohol and its hemolymph was collected by puncturing the ventral foot with sterile acupuncture. Each larva was collected only once.

### 2.4. Determination of DDC Activity and Dopamine Content

Sample processing: Fresh hemolymph was immediately boiled for 2 min to terminate enzymatic activity and centrifuged at 4000× *g* for 10 min at 4 °C to remove cellular debris. Chloroform was added and mixed by vortex and then centrifuged at 12,000 rpm for 10 min at 4 °C. The aqueous phase was collected and filtered through a 0.22 μm membrane for high-performance liquid chromatography (HPLC) with a C18 column.

DOPA detection: The mobile phase consisted of methanol and 0.1% (*v*/*v*) aqueous acetic acid (20:80, *v*/*v*), delivered at a flow rate of 1.0 mL/min. DOPA eluted at approximately 14 min and exhibited a characteristic absorbance maximum at 283 nm. The calibration curve demonstrated excellent linearity (y = 0.3231x − 0.01745, R^2^ = 0.99803; Figure S1).

Dopamine detection: The mobile phase employed acetonitrile/phosphate buffer (2:98, *v*/*v*) at a flow rate of 0.4 mL/min. Dopamine eluted at approximately 21 min and exhibited a characteristic absorbance maximum at 287 nm. Quantification was based on a linear calibration curve (y = 4.40 × 10^5^x − 1.28 × 10^4^, R^2^ = 0.99977; Figure S2).

DDC catalyzes the conversion of DOPA to dopamine. DDC activity was calculated based on the relative conversion of DOPA to dopamine in hemolymph samples. Each sample comes from at least 10 larvae and is tested three times. Each group has 3 samples.

### 2.5. Determination of PO Activity

Fresh hemolymph and PBS were mixed at a ratio of 1:3 (*v*/*v*), and then centrifuged at 13,000× *g* for 10 min at 4 °C to remove hemocyte. L-DOPA was added to the final concentration at 0.01 M and then briefly mixed (approximately 3 s) to measure absorbance at 490 nm. Absorbance was recorded every minute for 60 min. PO activity was calculated based on the rate of increase (ΔA490/min). Each group consists of three independent samples, with each hemolymph sample from at least 10 larvae, and each sample undergoes three repeated technical measurements. The average of the results is taken.

### 2.6. Melanization Assay

Next, 2 μL *E. coli* and 8 μL inhibitor were added into 200 μL of fresh hemolymph, and then immediately sealed and observed for 24 h at 26 °C.

Add 20 μL of the aforementioned fresh hemolymph mixture into 180 μL of PBS to incubate at 37 °C for 30 min, and then measure absorbance at 490 nm using a microplate reader (Bio Rad, Hercules, CA, USA). Each sample was analyzed three times as a technical replica.

Each group had three repeated samples. The control is PBS-replacing inhibitors under the same conditions.

In RNAi experiments, injection of dsRNA was followed by injection of *E. coli* 36 h later, and larvae hemolymph was collected for observation 6 h later to measure absorbance at 490 nm. The control group was injected with dsEGFP under the same conditions.

### 2.7. Antibacterial Activity Assay

Next, 2 μL of *E. coli* and 8 μL of inhibitor were added to 200 μL of fresh hemolymph, and then incubated at 26 °C for 3 h. The mixed solution was diluted 10^8^-fold in PBS, then 100 μL was spread onto LB agar plates to incubate overnight at 37 °C for colony-forming units. Each group has 3 portions of hemolymph mixture, and each hemolymph sample is coated on 3 plates. Control is PBS-replacing inhibitors under the same conditions.

In RNAi experiments, hemolymph was collected 36 h after injection of dsRNA, and only 2 μL of *E. coli* was added to incubate.

### 2.8. Hemocyte Aggregation Assay

After injecting inhibitor, the larvae were injected with *E. coli*. After 3 h, the larvae hemolymph was collected and observed under a microscope (Leica DM6 B, Leica Microsystems, Wetzlar, Germany) on a hemocytometer. A cell mass is defined as a cluster containing at least three cells under vertical view. The areas of hemocyte aggregation were counted using ImageJ software (version 1.54p) from five horizons (center, upper left, upper right, lower left and lower right), and the total areas were the sum of five horizons. Each group has 3 hemolymph samples, and each sample has 3 hemocytometers. Each hemocytometer was equipped with 10 μL of hemolymph. The control is PBS-replacing inhibitors under the same conditions.

In RNAi experiments, injection of dsRNA was followed by injection of *E. coli* 36 h later, and larvae hemolymph was collected for observation 6 h later.

### 2.9. Hemocyte Phagocytosis or Encapsulation Assay

Fresh hemolymph was immediately mixed with an equal volume of ice-cold anticoagulant buffer (186 mM NaCl, 41 mM citric acid, 98 mM NaOH and 17 mM EDTA with a few crystals of phenylthiourea, pH 4.5). The mixed solution was centrifuged at 800× *g* for 5 min at 4 °C to remove plasma, and then washed with PBS. The isolated hemocytes were resuspended in Grace medium at a density of 5–6 × 10^6^/mL. Next, 2 μL of *E. coli* and 8 μL of inhibitor were added to 200 μL of hemocytes solution and then incubated at 26 °C for 3 h. The incubated solution was observed under a fluorescence microscope after quenching the extracellular fluorescence with trypan blue. The control is PBS-replacing inhibitors under the same conditions. The data collection method for recording bacterial count based on fluorescence was the same as measuring the level of cell aggregation.

Ni-NTA agarose beads were suspended in Grace medium to a density of approximately 10^3^/μL after washing successively using TBS (PH 7.2) and Grace medium. Ni-NTA solution replaced *E. coli* to incubate and the incubated solution was observed under a microscope for encapsulation assay. The amount of hemocytes adhered on Ni-NTA agarose beads were estimated according to the method of measuring cell aggregation level.

### 2.10. Double-Stranded RNA Synthesis

The dsRNAs were synthesized according to the instructions of the T7 RibomaxTM Express RNAi System kit (Promega, Madison, WI, USA). Integrity of dsRNA was confirmed by nondenaturing agarose gel electrophoresis. Primers for the dsRNA synthesis of *BmDDC* were based on 600–1400 bp of the ORF and amplified a 653 bp product. Primers for the dsRNA synthesis of *BmPPO1* were based on sequences from 1000 to 2300 bp of the ORF and the length of the fragment amplified was 953 bp. Primers for the dsRNA synthesis of *BmPPO2* were based on sequences from 600 to 1900 bp of the ORF and the length of the fragment amplified was 1175 bp (Table S1). The concentration of dsRNA fragments was adjusted to 7 ng/μL.

### 2.11. Reverse Transcription-qPCR (RT-qPCR)

Total RNA was extracted from silkworm hemolymph using a commercial RNA extraction kit (Takara, Beijing, China) according to the manufacturer’s instructions. cDNA synthesis was performed using the ReverTra Ace qPCR RT Kit (Toyobo, Shanghai, China) following the manufacturer’s protocol. RT-qPCR primers were designed with Primer 5.0 software based on the ORF sequences of *BmPPO1*, *BmPPO2*, and *BmDDC* (Table S1). Primers targeting *BmTif4A* were used as an internal control [40] (Table S1).

### 2.12. Nodule Formation Assay

Larvae with healthy phenotypes were dissected under a stereomicroscope (Zeiss SteREO Discovery V8, Carl Zeiss AG, Oberkochen, Germany), and melanized nodules in the hemocoel were counted. Nodules were defined as discrete, darkly pigmented hemocyte aggregates larger than 50 μm in diameter. Dissect at least five larvae per group.

### 2.13. Data Analysis

All data are presented as mean ± standard error of the mean (SEM). Statistical analyses were conducted using IBM SPSS Statistics (version 27). For comparisons between two groups, an unpaired two-tailed Student’s *t*-test was used after confirming normal distribution and homogeneity of variance.

## 3. Results

### 3.1. Specificity of Inhibitor

**Figure 1:**
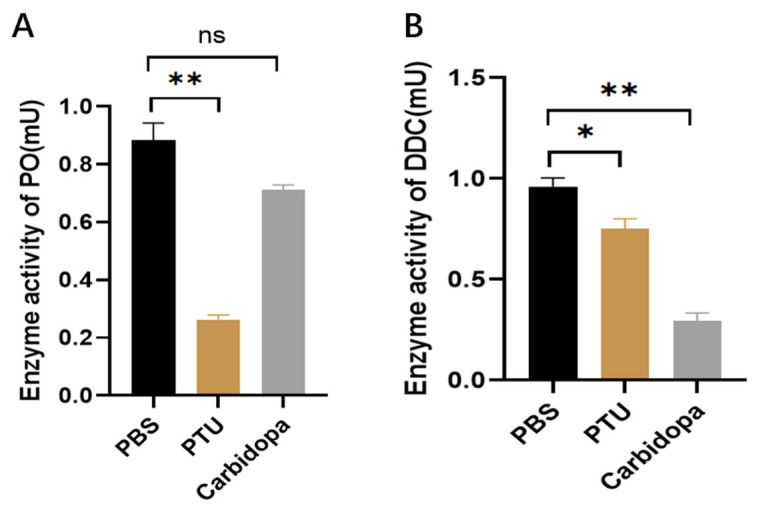
Effects of inhibitors on PO and DDC enzymatic activity. (**A**) Measurement of PO enzyme activity following treatment with PBS, PTU, and carbidopa. (**B**) Measurement of DDC enzyme activity following treatment with PBS, PTU, and carbidopa. * *p* < 0.05, ** *p* < 0.01; ns indicates no significant difference (*p* ≥ 0.05) by independent samples *t*-test.

To investigate the roles of PO and DDC in hemolymph immune melanization, we assessed the specificity of their respective inhibitors. PO activity was analyzed by adding dopa into the hemolymph-containing inhibitor, then measuring the absorbance at 490 nm. In contrast, the inhibitory effects on DDC activity were assessed after injection, and dopa and dopamine levels in the larval hemolymph were quantified by HPLC. PTU treatment resulted in approximately a 70% reduction in PO activity, whereas carbidopa caused a slight decrease that was not statistically significant (Figure 1A). In contrast, DDC activity was reduced by 32% in the PTU-treated group and by 74% following carbidopa treatment (Figure 1B). These results indicate that carbidopa specifically inhibits DDC activity, whereas PTU mainly acts as an inhibitor of PO.

### 3.2. Effect of Inhibitors on the Melanization and the Antimicrobial Activity of Hemolymph

**Figure 2:**
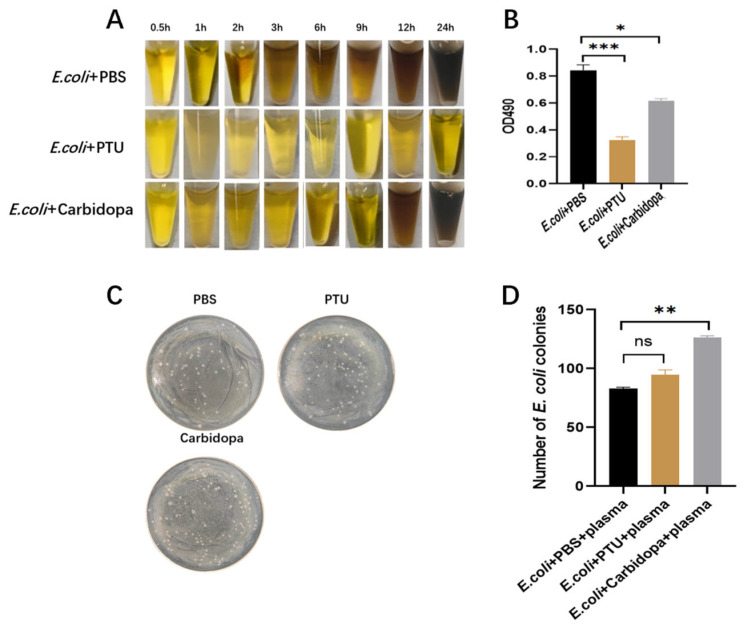
Analysis of inhibitors on the melanization and the antimicrobial activity of hemolymph. (**A**) Melanization of hemolymph following treatment with *E. coli* + PBS, *E. coli* + PTU, or *E. coli* + carbidopa. (**B**) Measurement of hemolymph absorbance following treatment with *E. coli* + PBS, *E. coli* + PTU, or *E. coli* + carbidopa. (**C**) Colony growth after treatment with PBS, PTU, or carbidopa. (**D**) Colony counts after treatment with *E. coli* + PBS + plasma, *E. coli* + PTU + plasma, or *E. coli* + carbidopa + plasma. * *p* < 0.05, ** *p* < 0.01, *** *p* < 0.001; ns indicates no significant difference (*p* ≥ 0.05) by independent samples *t*-test.

To evaluate the effects of PTU and carbidopa on hemolymph melanization, hemolymph was incubated with *E. coli* and inhibitor in sealed microcentrifuge tubes, and melanization was monitored over time. In the control group, hemolymph melanization was evident within 1 h and progressively intensified thereafter. In contrast, the treatment group with carbidopa showed a significant delay in melanin formation, with visible melanin formation only observed after approximately 6 h of incubation. Notably, no apparent melanization was detected in the PTU-treated group throughout the entire observation period (Figure 2A). Consistent with these visual observations, the absorbance analysis further indicated that compared to the control group, the hemolymph absorbance values of the carbidopa and PTU treatment groups were significantly reduced. The inhibitory effect was more pronounced in the PTU-treated group than in the carbidopa-treated group (Figure 2B). Overall, these results indicate that both PO and DDC contribute to hemolymph melanization during the immune response, with PO showing a more prominent effect on the oxidation-associated absorbance signal.

To further evaluate the effects of PTU and carbidopa on the antibacterial activity of hemolymph, an antibacterial assay was performed. Compared with the PBS group, the carbidopa-treated group exhibited an approximately 20% increase, and the PTU-treated group showed a slight increase in bacterial colony counts (Figure 2C,D). These results suggest that both PO and DDC activities contribute to the antibacterial capacity of hemolymph, with DDC exerting a stronger impact.

### 3.3. Effects of Inhibitors on Cellular Responses (Aggregation, Phagocytosis, Encapsulation) and Dopamine Titers

**Figure 3:**
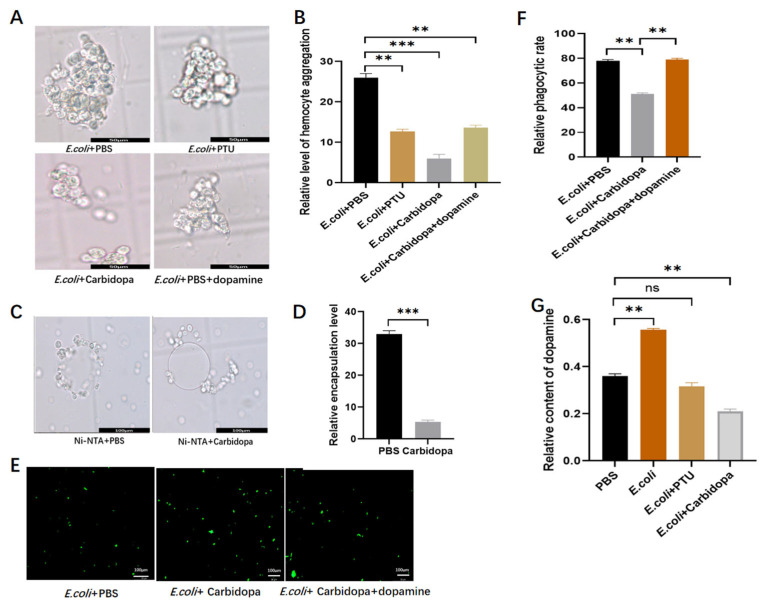
Analysis of inhibitor effects on cellular immune responses and hemolymph dopamine levels. (**A**) Morphological changes of hemocytes after treatment with *E. coli* + PBS, *E. coli* + PTU, *E. coli* + carbidopa, or *E. coli* + PBS + dopamine. (**B**) Quantification of hemocyte aggregation after treatment with *E. coli* + PBS, *E. coli* + PTU, *E. coli* + carbidopa, or *E. coli* + carbidopa + dopamine. (**C**) Hemocyte encapsulation of Ni–NTA agarose beads following treatment with PBS or carbidopa in vitro. (**D**) Quantification of hemocyte encapsulation after treatment with PBS and carbidopa. (**E**) Fluorescent images showing phagocytosis of *E. coli* by hemocytes following treatment with *E. coli* + PBS, *E. coli* + carbidopa, or *E. coli* + carbidopa + dopamine. (**F**) Quantification of hemocyte phagocytic activity (devouring rate) after treatment with *E. coli* + PBS, *E. coli* + carbidopa, or *E. coli* + carbidopa + dopamine. (**G**) Dopamine concentration in hemolymph determined by HPLC following treatment with PBS, *E. coli*, *E. coli* + PTU, or *E. coli* + carbidopa. ** *p* < 0.01, *** *p* < 0.001; ns indicates no significant difference (*p* ≥ 0.05) by independent samples *t*-test.

To further examine the roles of PO and DDC in cellular immune responses, the larvae were injected with the inhibitor and subsequently challenged with *E. coli*. Hemocyte morphology and melanization were then analyzed. In the PBS-treated control group, hemocytes exhibited extensive aggregation and overlapping, forming large cellular clusters in which melanized hemocytes were frequently observed (Figure 3A). In contrast, carbidopa treatment markedly reduced both the number and size of hemocyte aggregates, and most hemocytes remained dispersed (Figure 3A,B). In the PTU-treated group, hemocyte aggregation was greater than that observed in the carbidopa-treated group but slightly reduced compared with the control. Notably, melanized hemocytes were not detected in either the carbidopa- or PTU-treated groups, consistent with the previously observed inhibition of hemolymph melanization. Addition of dopamine after carbidopa treatment restored the formation of large hemocyte clusters in the hemolymph (Figure 3A). To assess hemocyte encapsulation activity, isolated hemocytes were incubated with Ni-NTA agarose beads in the presence of carbidopa. In the PBS-treated group, numerous hemocytes adhered to and encapsulated the agarose beads (Figure 3C). In contrast, carbidopa treatment significantly reduced hemocyte attachment, and the encapsulation area decreased to approximately 15% of that in the control group (Figure 3D). Hemocyte phagocytic activity was further examined using fluorescence microscopy. Compared with the PBS-treated group, fluorescence intensity in the carbidopa-treated group was significantly increased by approximately 20% (Figure 3E,F). When dopamine was simultaneously added together with carbidopa, the fluorescence intensity showed no significant difference from that of the control group (Figure 3E,F). Dopamine levels in larval hemolymph were quantified by HPLC. Injection of *E. coli* resulted in a significant increase in dopamine concentration, whereas PTU treatment reduced dopamine levels to those observed in the PBS-treated group (Figure 3G). In contrast, carbidopa treatment significantly decreased dopamine levels to approximately 50% of those in the PBS group (Figure 3G). Taken together, these results indicate that both DDC and PO participate in hemocyte-mediated immune responses associated with melanization. However, inhibition of DDC produced stronger effects on several cellular immune parameters, which was consistent with the results of the previous colony growth experiment.

### 3.4. Effects of Reducing Gene Expression on the Antibacterial Activity and Melanization of Hemolymph

**Figure 4:**
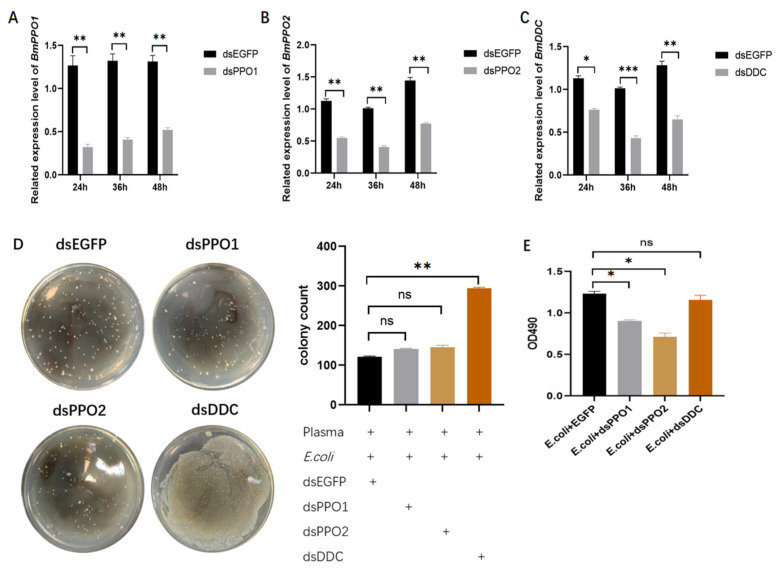
Gene silencing effects on hemolymph antibacterial activity and melanization. (**A**–**C**) RT–qPCR analysis of gene silencing efficiency of *BmPPO1* (**A**), *BmPPO2* (**B**), and *BmDDC* (**C**) in hemolymph at 24, 36, and 48 h after dsRNA injection. (**D**) Colony growth plates and quantitative analysis of plaque counts in hemolymph from larvae injected with dsEGFP, dsBmPPO1, dsBmPPO2, or dsBmDDC. (**E**) Absorbance analysis reflecting the extent of melanization following treatment with *E. coli* + EGFP, *E. coli* + DsPPO1, *E. coli* + dsPPO2, or *E. coli* + dsDDC. * *p* < 0.05, ** *p* < 0.01, *** *p* < 0.001; ns indicates no significant difference (*p* ≥ 0.05) by independent samples *t*-test.

DsRNA targeting *BmPPO1*, *BmPPO2*, or *BmDDC* (dsBmPPO1, dsBmPPO2, or dsBmDDC) was injected into larvae, with dsRNA targeting *EGFP* (dsEGFP) used as a control. Gene silencing efficiency in the hemolymph was evaluated at 24, 36, and 48 h after injection by qRT–PCR. All three genes exhibited maximal knockdown efficiency at 36 h post-injection (Figure 4A–C). Based on these results, hemolymph samples were collected 36 h after dsRNA injection and subjected to antibacterial activity assays. Compared with the dsEGFP control group, plaque counts in the dsBmDDC groups showed a significant increase (Figure 4D), indicating that reduction of *BmDDC* expression significantly decreased the antibacterial activity of hemolymph. Knockdown of *BmPPO1* or *BmPPO2* individually also showed a similar trend, although the differences were not statistically significant. To further investigate the effects of gene silencing on hemolymph melanization, larvae were challenged with *E. coli* 36 h after dsRNA injection. Hemolymph was collected 6 h later to measure absorbance. Reduction of *BmPPO1* or *BmPPO2* expression led to a significant decrease in hemolymph absorbance, with *BmPPO2* knockdown showing a more pronounced effect (Figure 4E). In contrast, reduction of *BmDDC* expression did not result in a statistically significant change in absorbance (Figure 4E). Together, these results suggest that suppression of PPO genes has a stronger impact on melanization-associated absorbance, consistent with previous inhibitor experiment results.

### 3.5. Effects of Reducing Gene Expression on Hemocyte Aggregation and Epidermal Nodule Formation

**Figure 5:**
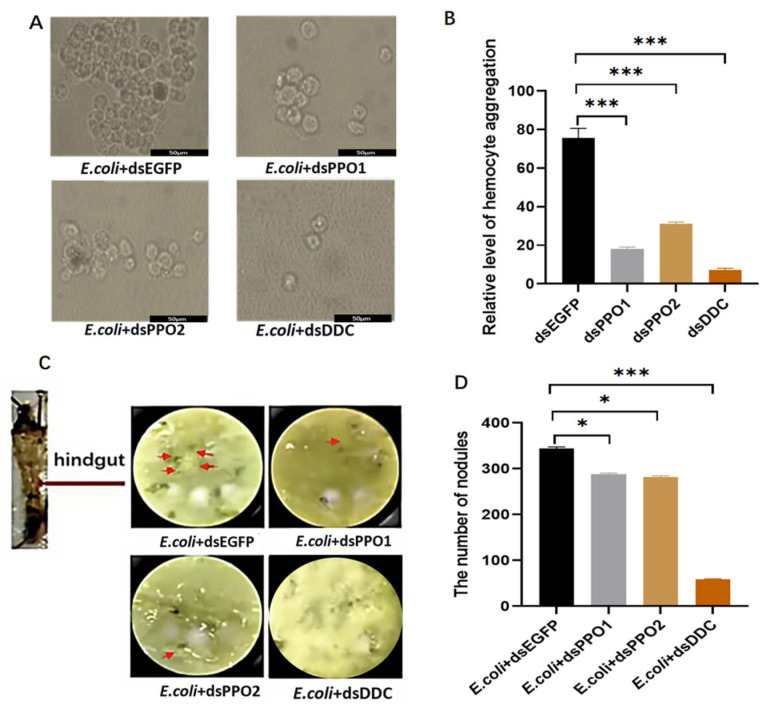
Analysis of hemocyte aggregation and nodule formation after dsRNA injetion. (**A**) Hemocyte morphology following treatment with *E. coli* + dsEGFP, *E. coli* + dsPPO1, *E. coli* + dsPPO2, or *E. coli* + dsDDC. (**B**) Quantification of the extent of hemocyte aggregation following treatment with dsEGFP, dsPPO1, dsPPO2, or dsDDC. (**C**) Number of melanized nodules following treatment with *E. coli* + dsEGFP, *E. coli* + dsPPO1, *E. coli* + dsPPO2, or *E. coli* + dsDDC. (**D**) Nodule numbers after treatment with *E. coli* + dsEGFP, *E. coli* + dsPPO1, *E. coli* + dsPPO2, or *E. coli* + dsDDC. * *p* < 0.05, *** *p* < 0.001.

At 36 h following dsRNA treatment, hemocyte morphology was assessed in larvae that had been injected with *E. coli*. In the dsEGFP control group, large numbers of hemocytes aggregated to form prominent cellular clusters, within which a small proportion of melanized cells was observed (Figure 5A,B). In contrast, both the number and size of hemocyte aggregates were significantly reduced in the dsBmPPO1, dsBmPPO2, and dsBmDDC groups. The levels of aggregation in these groups were approximately 15.5%, 29.7%, and 5.7%, respectively, of those observed in the control group (Figure 5A,B). These results indicate that reduction of *BmDDC*, *BmPPO1*, or *BmPPO2* expression impairs hemocyte aggregation, with *BmDDC* knockdown exerting the most pronounced effect. To assess nodule formation, larvae were dissected and fat bodies were removed to visualize melanized nodules (Figure 5C,D). The numbers of nodules were 329 in the dsEGFP group, 66 in the dsBmDDC group, 282 in the dsBmPPO1 group, and 277 in the dsBmPPO2 group (Figure 5C,D). These data indicate that knockdown of *BmPPO1* or *BmPPO2* results in a modest reduction in nodule formation, whereas reduction of *BmDDC* expression markedly suppresses nodule formation.

## 4. Discussion

Melanization represents a fundamental and evolutionarily conserved defense mechanism in arthropods, playing a critical role in immune responses against a wide range of pathogens, including bacteria, fungi, parasites, and viruses [8,33,42,43,44,45,46]. Hemolymph melanization is primarily mediated by enzymes involved in melanin biosynthesis, among which PO and DDC are key components [28,29,47,48,49]. In the present study, considering these enzymes function within an interconnected melanization cascade, we conducted an assessment of their relative contributions to melanin production, antibacterial activity, and hemocyte-mediated immune responses. Inhibition of either PO or DDC activity significantly delayed hemolymph melanization following *E. coli* infection, with PTU treatment producing a more pronounced effect. Consistently, RNAi-mediated knockdown of *BmPPO1* or *BmPPO2* significantly reduced hemolymph absorbance associated with melanization, further supporting the prominent role of *BmPPO* in melanin formation. Together, these results indicate that both PO and DDC contribute to hemolymph melanization, although PO activity has a stronger influence on the extent of melanin formation. Although PO and DDC jointly participate in melanization, their relative contributions to antibacterial defense appear to differ. Pharmacological inhibition of DDC by carbidopa, as well as RNAi-mediated reduction of *BmDDC* expression, resulted in a significant decrease in hemolymph antibacterial activity. In contrast, inhibition of PO activity by PTU or knockdown of *BmPPO1* or *BmPPO2* also reduced antibacterial capacity, but the effect was less pronounced than that observed following DDC suppression. These findings suggest that DDC may influence antibacterial defense not only by regulating substrate availability for PO-mediated melanization chemistry, but also through additional immune-related processes associated with catecholamine metabolism.

Cellular immune responses constitute a central component of insect innate immunity, encompassing processes such as hemocyte aggregation, encapsulation, phagocytosis, and nodulation, which collectively facilitate the clearance of invading pathogens from the hemolymph [50,51,52,53,54,55]. Consistent with previous reports in silkworms [33], we observed extensive hemocyte aggregation following *E. coli* challenge, with hemocytes forming large multicellular clusters in the hemocoel. Both pharmacological inhibition and RNAi-mediated gene silencing resulted in reduced hemocyte aggregation, phagocytosis, encapsulation of Ni-NTA agarose beads, and nodule formation. Suppression or knockdown of *BmDDC* produced more pronounced effects on these cellular immune responses, although inhibition of PO also caused measurable reductions. Notably, restoration of PO substrate availability partially rescued the impaired immune phenotypes. Dopamine is produced and released by immune cells and has been implicated in the regulation of hemocyte behavior and immune competence [29,32,56,57,58,59]. These results suggest that DDC plays a relatively prominent role in regulating hemocyte-mediated cellular immune responses. This effect may arise because DDC regulates the availability of physiological substrates required for PO-dependent melanization cascades. These results seem to differ from but support the current understanding that DHI and PO generate reactive intermediates with high cytotoxicity and broad-spectrum antibacterial activity, which are the main reasons for cellular immune responses and antibacterial defense. In addition, our findings are consistent with studies in various arthropods, including *C. capitata*, *Litopenaeus vannamei*, and *A. subalbatus* [29,30,32,60]. DDC contributes broadly to multiple facets of hemocyte function, extending beyond its canonical role in melanin synthesis [32,61]. In addition, Wang et al. reported that melanization rates in *Spodoptera exigua* larvae were closely correlated with PO or DDC activity, whereas pathogen load was significantly associated with DDC activity rather than PO activity [62].

While our study provides insight into the relative contributions of *BmPPO* and *BmDDC* in hemolymph melanization and immune defense in *B. mori*, several limitations should be acknowledged. First, only a single bacterial species (*E. coli*) was used as the immune stimulus, which may limit the generalization of the findings to other pathogens. Previous studies, including our preliminary work, have shown that multiple microbes and their cell wall components can induce hemolymph melanization; however, for practical and biosafety reasons, we focused on *E. coli* in this study [36]. Second, the experiments primarily examine phenotypic outcomes, such as hemolymph melanization absorbance and hemocyte responses, without direct biochemical characterization of intermediate oxidation products. Finally, although RNAi and pharmacological manipulations allowed us to evaluate the relative contributions of *BmPPO* and *BmDDC*, the molecular mechanisms underlying them remain to be fully elucidated.

Compared with hemocoelic injections, oral RNAi is less invasive and more convenient. However, in lepidopteran insects such as *B. mori*, oral RNAi has historically shown limited efficiency, primarily due to dsRNA degradation in the gut lumen, poor cellular uptake, and systemic transport barriers. Recent advances have partially overcome these limitations through the development of nanocarrier-based delivery systems. Various nanoparticles, including chitosan-based nanoparticles, liposomes, and other polymeric complexes, have been shown to protect dsRNA from degradation and enhance its uptake across the gut epithelium. Notably, successful implementation of oral RNAi in *B. mori* has been reported using nanoparticle-mediated delivery, demonstrating improved gene silencing efficiency and expanding the applicability of RNAi in this species [41,63,64,65]. These technological developments highlight the potential of oral RNAi as a complementary or alternative strategy to injection-based approaches.

## 5. Conclusions

Our study reveals the relative contributions of DDC and PO within immune melanization of insect hemolymph. Both enzymes influence melanization intensity as well as antibacterial and cellular immune responses. Restoration of PO substrate (as dopamine) availability partially rescued the defects caused by DDC inhibition. Specifically, PO appears to contribute predominantly to melanin production, whereas DDC exerts additional effects on antibacterial and cellular immune responses.

## Data Availability

The original contributions presented in this study are included in the article/Supplementary Materials. Further inquiries can be directed to the corresponding author.

## Supplementary Materials

The following supporting information can be downloaded at: https://www.mdpi.com/article/10.3390/insects17040405/s1, Table S1. Sequences of primers used in this study; Figure S1. (A) Chromatogram of dopamine standard; (B) UV spectrum of dopamine standard; (C) Standard calibration curve of dopamine; (D) Chromatogram of dopamine sample. The experiment used high-performance liquid chromatography with a fluorescence detector (HPLC-FLD) to measure DDC enzyme activity. In subfigure (A), under the elution of mobile phase acetonitrile: phosphate solution = 2:98, the standard dopamine substrate showed a peak at approximately 21 min. Subfigure (B) shows that the UV spectrum also exhibited a maximum at 287 nm for this peak. Subfigure (C) presents the linear regression curve as y = 440174.83379x (±3365.14829) − 12767.89411 (±25893.79017), R^2^ = 0.99977. The sample results are shown in subfigure (D); compared with the control group, injection of *E. coli* significantly increased dopamine content; Figure S2. (A) Chromatogram of L-DOPA standard; (B): UV spectrum of L-DOPA standard; (C) Standard calibration curve of L-DOPA; (D) Chromatogram of L-DOPA sample. HPLC was used to determine the DDC enzyme activity in silkworms. Larvae were injected in the hemocoel with the PO inhibitor phenylthiourea and the DDC inhibitor carbidopa, and hemolymph was collected for analysis 0.5 h later. As shown in subfigure (A), the mobile phase was methanol: acetic acid water (20:80), and the dopamine standard eluted at 14 min. In subfigure (B), the UV detection wavelength was 283 nm. Subfigure (C) shows the standard curve with the regression equation y = 0.3231x (±0.00641) − 0.01745 (±0.0457), R^2^ = 0.99803. Subfigure (D) shows the sample chromatogram.
