# Supplementary material for: The Relative Contributions of BmPPO and BmDDC in Immune Melanization of Hemolymph in Silkworm, Bombyx mori
Source: Insects. 2026 Apr 9;17(4):405. doi: 10.3390/insects17040405 (PMC13115849; doi:10.3390/insects17040405)
Supplement: Supplementary file 1 [file insects-17-00405-s001.zip › insects-4182441-supplementary.pdf]

Supplementary tables

Table S1. Sequences of primers used in this study

Primer sequence

Name	Sequences（5'→3'）
BmTif4	F：TTCGTACTGGCTCTTCTCGT
	R：CAAAGTTGATAGCAATTCCCT
BmPPO1	F：CCCTACTACGGCGACCTCCACA
	R：GCTGGCGGGTCCTGTCGGTGAA
BmDDC	F：GCTAAAATCACTACAGCCAGAC
	R：GTTTATACGGCGT AATAGTTCTT
BmPPO2	F：TTATCACTGGCATTTGGTCTAC
	R：AACATAACAAACAGTTGGAAGG
dsBmDDC	F：TAATACGACTCACTATAGGGGCTAAAATCACTACAGCCAGAC
	R：TAATACGACTCACTATAGGGGTTTATACGGCGTAATAGTTCTT
dsBmPPO1	F：TAATACGACTCACTATAGGGCCCTACTACGGCGACCTCCACA
	R：TAATACGACTCACTATAGGGGCTGGCGGGTCCTGTCGGTGAA
dsBmPPO2	F：TAATACGACTCACTATAGGGTTATCACTGGCATTTGGTCTAC
	R：TAATACGACTCACTATAGGGAACATAACAAACAGTTGGAAGG
qBmTif4	F：TTCGTACTGGCTCTTCTCGT
	R：CAAAGTTGATAGCAATTCCCT
qBmPPO1	F：CCCTACTACGGCGACCTCCACA
	R：GCTGGCGGGTCCTGTCGGTGAA
qBmDDC	F：GCTAAAATCACTACAGCCAGAC
	R：GTTTATACGGCGT AATAGTTCTT
qBmPPO2	F：TTATCACTGGCATTTGGTCTAC
	R：AACATAACAAACAGTTGGAAGG

Supplementary figures

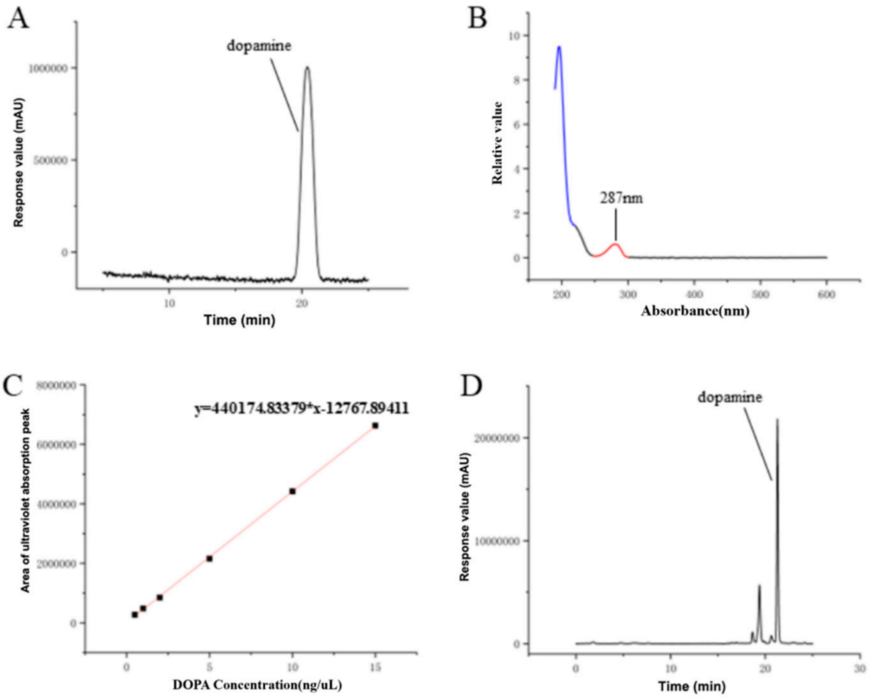

Figures S1. A: Chromatogram of dopamine standard; B: UV spectrum of dopa mine standard; C: Standard calibration curve of dopamine;D: Chromatogram of dopamine sample;

The experiment used high-performance liquid chromatography with a fluorescence detector (HPLC-FLD) to measure DDC enzyme activity. In Figure A, under the elution of mobile phase acetonitrile: phosphate solution = 2:98, the standard dopamine substrate showed a peak at approximately 21 minutes. Figure B shows that the UV spectrum also exhibited a maximum at 287 nm for this peak. Figure C presents the linear regression curve as y = 440174.83379x (±3365.14829) - 12767.89411 (±25893.79017), R^2 = 0.99977. The sample results are shown in Figure D; compared with the control group, injection of E. coli significantly increased dopamine content.

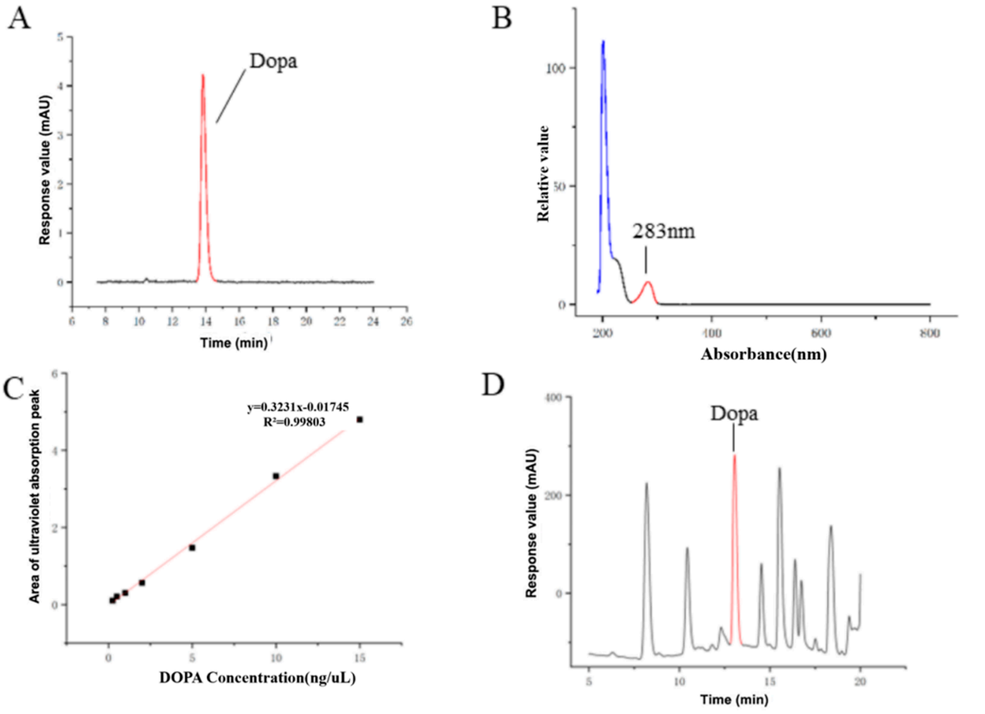

Figures S2. A: Chromatogram of L-DOPA standard; B: UV spectrum of L-DOPA standard; C: Standard calibration curve of L-DOPA; D: Chromatogram of L-DOPA sample;

HPLC was used to determine the DDC enzyme activity in silkworms. Larvae were injected in the hemocoel with the PO inhibitor phenylthiourea and the D DC inhibitor carbidopa, and hemolymph was collected for analysis 0.5 hours la ter. As shown in Figure A, the mobile phase was methanol: acetic acid water (20:80), and the dopamine standard eluted at 14 minutes. In Figure B, the UV detection wavelength was 283 nm. Figure C shows the standard curve with th e regression equation $y = 0.3231x\ (\pm 0.00641) - 0.01745\ (\pm 0.0457)$, $R^2 = 0.9$ 9803. Figure D shows the sample chromatogram.
